# In the literature: April 2021

**DOI:** 10.1016/j.esmoop.2021.100116

**Published:** 2021-04-20

**Authors:** P. Martín-Martorell, I. Gonzalez-Barrallo, V. Gambardella, J.M. Cejalvo, A. Cervantes

**Affiliations:** 1 Department of Medical Oncology, Biomedical Research Institute INCLIVA, University of Valencia, Valencia, Spain; 2 CIBERONC, Instituto de Salud Carlos III, Madrid, Spain

## Patterns of transcription factor programs and immune pathway activation in small-cell lung cancer

Small-cell lung cancer (SCLC) remains elusive to significant treatment advances despite the recent incorporation of immune checkpoint blockade (ICB) to treatment algorithms. The recognition of distinct neuroendocrine (NE) transcription factor-based SCLC variants (*ASCL1*, *NEUROD1*, *POUF3,* and *YAP1*) by Rudin and Zhang has identified disease subsets with potentially different therapeutic vulnerabilities. However, the intertumoral and intratumoral heterogeneity have not been yet fully explored, and non-NE subsets POUF3 and specially YAP1 do not seem homogenous entities with fully concordant immunohistochemistry (IHC) protein expression.^1^^,^^2^

In their recently published article, Gay and colleagues^3^ nicely attempt to clarify these heterogeneous non-NE subsets and the mechanisms underlying platinum resistance. Through a negative matrix factorization approach, they identify a subset of SCLC without a distinct transcriptional signature, but with a clear predominance of immune- and HLA-related gene expression and interferon-γ activation which they call SCLC-inflamed (SCLC-I), as well as low IHC expression of ACSL1, NEUROD1, and POUF3 which, on the other hand, does not necessarily correlate with YAP1 overexpression or its transcriptional targets.^3^ This was validated in an LS-SCLC cohort and also in an ES-SCLC cohort from the IMpower133 trial, the first randomized trial to demonstrate overall survival (OS) improvement with ICB in SCLC.^4^

They corroborate a higher IHC expression of chromogranin and synaptophysin NE markers in SCLC-A and SCLC-N subtypes, while an epithelial–mesenchymal transition (EMT)-associated phenotype showing vimentin and AXL overexpression was preferentially associated with the SCLC-I subset. No significant differences were found with regard to *RB1* and *TP53* gene expression across the subsets, and only *MYC* showed a preferential overexpression in the POUF3 expressing (SCLC-P) subtype. Through the integration of proteomic, genomic, and transcriptomic data, they identify distinct phenotypic groups. *ASCL1* overexpressing (SCLC-A) is an NE, epithelial subtype characteristic of TTF1-positive SCLC. While still highly NE, the *NEUROD1* overexpressing subset (SCLC-N) largely lacks TTF1 expression. Non-NE SCLC consists of SCLC-P and SCLC-I, which can be further subdivided based on EMT features. By applying a broad drug screen augmented sensitivity to PARP inhibitors was seen in the SCLC-P subset, while SCLC-A models were highly sensitive to Aurora kinase inhibitors. Histone deacetylase inhibitors were capable of reversing vimentin and augmenting E-cadherin expression in SCLC-I models, consistent with EMT reversal.

Gay et al. also very neatly explore intratumoral heterogeneity of SCLC and its relevance in platinum-resistance development. Using single-cell RNA sequencing methodology, they show that, while most cells indeed preferentially express only one of the transcription factors, this is not fully mutually exclusive and that this can occur at single-cell level. They show the emergence of a differential island cluster upon resistance of predominant platinum-sensitive ASCL1 models, and this cluster to be characterized by ASCL1-negative cells which remain negative for NEUROD1, POUF3, and YAP1 expression. This cluster, however, notably gains expression of MHC class II and EMT-related genes, concordant with an SCLC-I genotype shift. Their observations support a high plasticity of the emerging cluster population. They show this population change upon resistance to be the result of a gene expression shift at single-cell level, rather than the result of a proliferative effort of resistant cell clusters.

We could not agree more with the last sentences of the authors of this inspiring article. Most SCLC tumors and models analyzed were easily classified into one of the four subtypes, permitting a realistic scenario in which prospective subtyping is performed in a single umbrella trial, wherein patients are assigned to a treatment arm (SCLC-I to combination ICB, SCLC-P to PARP inhibitors) on the basis of their SCLC subtype. Subtype could be determined, and then monitored dynamically, by transcriptional, proteomic, or even epigenetic classification. If any one of these predictions demonstrated significant benefit, it would represent the first standard-of-care molecular biomarker selection for SCLC and a foundational step toward personalized therapy for this devastating disease.

## The relevance of pathological response after neoadjuvant therapy in stage III melanoma as a surrogate of survival

The recent approval of immunotherapy and BRAF/MEK inhibitors in the adjuvant setting has revolutionized adjuvant therapy for melanoma patients, representing a significant breakthrough. However, despite the introduction of an active adjuvant approach, the prognosis of stage III melanoma remains poor. In an interesting article recently published in Nature Medicine by the International Neoadjuvant Melanoma Consortium,^5^ the role of neoadjuvant treatment was evaluated in a pooled analysis including six neoadjuvant trials enrolling melanoma patients to receive anti-BRAF/MEK therapy or PD-1-based immunotherapy. The authors aimed to determine the impact of the pathologic response on the clinical outcomes with neoadjuvant therapy in patients with clinical stage III melanoma.

The analysis included 189 patients who underwent surgery on the primary tumors. All these patients were treated with neoadjuvant therapy (138 immunotherapy and 51 targeted therapy). The results demonstrated that neoadjuvant treatment has high antitumor activity, achieving a pathological complete response (pCR) in 75 (40%) patients, near pCR in 21 (11%), pathological partial response (pPR) in 27 (14%), and absence of pathological response in 66 (35%). In the multivariable analysis, the benefit was derived from the use of combined immunotherapy versus a single-agent approach. No baseline factor could be correlated with pCR. Interestingly, the degree of pathological response strongly correlates with both recurrence-free survival (pCR 2-year 89% versus no pCR 50%, *P* < 0.001) and OS (pCR 2-year OS 95% versus no pCR 83%, *P* = 0.027).

Among the two different neoadjuvant therapies, immunotherapy, independently from the degree of pathological response (pCR, near pCR, or pPR) obtained, improves survival, while, with the with use of targeted therapies, pCR was critical to confer it. However, as a general conclusion, neoadjuvant treatment appears to be more active than adjuvant with benefit in both relapse-free survival and OS. A limitation of this pooled analysis in evaluating the impact of pCR is the heterogeneity of the population in terms of the molecular characterization and the adjuvant approaches used. To avoid it, the International Neoadjuvant Melanoma Consortium is developing trials in which pathological response will be the primary endpoint,^6^ as it is an early surrogate endpoint of benefit. Neoadjuvant treatment could represent a step ahead for a more effective treatment in stage III melanoma.

## Direct and indirect regulators of epithelial–mesenchymal transition-mediated immunosuppression in breast carcinoma

ICB therapies provide significant survival benefits in many cancers. Different biomarkers for predicting responses have been described such as the presence of T-cells, PDL-1 expression, and tumor mutational burden.^7^ However, the efficacy of ICB varies considerably across different cancer types. In fact, it has not shown a wide success in treating breast cancer yet. Different mechanisms of resistance such as somatic mutations affecting carcinoma cell-intrinsic pathways associated with antigen-presentation and/or interferon-γ sensing have been described.

Dongre et al.^8^ published in Cancer Discovery a fascinating paper about the role of EMT in immunotherapy resistance. It is relevant to underline that tumors are essentially heterogeneous arising from mixtures of epithelial and quasi-mesenchymal carcinoma cells. First, they showed quasi-mesenchymal cells induced an immunosuppressive microenvironment. These cells regulated the recruitment of macrophages (M2-like) and T regulatory cells. Moreover, they induced a negative regulation of natural killer and T cells. By contrast, their epithelial counterpart recruited functional T cells and expressed immune genes associated with antigen presentation and cytokine secretion. This work demonstrated how a small subpopulation of quasi-mesenchymal cells can cross-protect their epithelial neighbor cells from antitumor immune attack. Second, they described that this antitumor immunity was strongly dependent on carcinoma cell-derived immunomodulatory paracrine factors.

Indeed, the regulation of these factors (CD73, CSF1, or SPP1) altered their susceptibility to antitumor immune attack. Besides, they proposed the clinical utility of perturbing the CD73–adenosine signaling axis to potentiate the efficacy of adoptive T-cell transfer therapy and ICB. Overall, the manuscript indicates that carcinoma cell-intrinsic factors specifically associated with the quasi-mesenchymal state can directly influence their response to ICB therapies. Therefore the authors suggest the possibility of using the epithelial–mesenchymal phenotypic states of carcinoma cells as a surrogate biomarker to predict responses to immunotherapy and as a plausible new target.
